# On the Efficient Evaluation of the Exchange Correlation Potential on Graphics Processing Unit Clusters

**DOI:** 10.3389/fchem.2020.581058

**Published:** 2020-12-10

**Authors:** David B. Williams-Young, Wibe A. de Jong, Hubertus J. J. van Dam, Chao Yang

**Affiliations:** ^1^Lawrence Berkeley National Laboratory, Computational Research Division, Berkeley, CA, United States; ^2^Brookhaven National Laboratory, Computational Science Initiative, Upton, NY, United States

**Keywords:** density functional theory, graphics processing unit, high-performance computing, parallel computing, quantum chemistry

## Abstract

The predominance of Kohn–Sham density functional theory (KS-DFT) for the theoretical treatment of large experimentally relevant systems in molecular chemistry and materials science relies primarily on the existence of efficient software implementations which are capable of leveraging the latest advances in modern high-performance computing (HPC). With recent trends in HPC leading toward increasing reliance on heterogeneous accelerator-based architectures such as graphics processing units (GPU), existing code bases must embrace these architectural advances to maintain the high levels of performance that have come to be expected for these methods. In this work, we purpose a three-level parallelism scheme for the distributed numerical integration of the exchange-correlation (XC) potential in the Gaussian basis set discretization of the Kohn–Sham equations on large computing clusters consisting of multiple GPUs per compute node. In addition, we purpose and demonstrate the efficacy of the use of batched kernels, including batched level-3 BLAS operations, in achieving high levels of performance on the GPU. We demonstrate the performance and scalability of the implementation of the purposed method in the NWChemEx software package by comparing to the existing scalable CPU XC integration in NWChem.

## 1. Introduction

Kohn-Sham density functional theory (KS-DFT) (Hohenberg and Kohn, 1964; Kohn and Sham, 1965) is unequivocally the computational workhorse of theoretical chemistry and materials science. With the excellent balance of its computational cost to its ability to accurately predict physical phenomena, KS-DFT is nearly without equal in the routine theoretical treatment of large, experimentally relevant systems (Ratcliff et al., 2017; Wu et al., 2019). A primary factor contributing to the popularity of KS-DFT methods is the existence of highly optimized and scalable software implementations capable of leveraging the latest advances in modern high-performance computing (HPC). The existence of such software enables the treatment of increasingly larger and more complicated systems as computing resources become large enough to accommodate them. Historically, these optimizations have amounted to considering the underlying details of homogeneous computing platforms such as shared and distributed memory multi-core central processing unit (CPU) architectures to exploit memory hierarchies, distributed node topology and interconnection, and computing features such as single-instruction multiple data (SIMD) instructions, fused multiply-add (FMA), etc. (Belling et al., 1999; Brown et al., 2008; Lasinski et al., 2008; de Jong et al., 2010; Bylaska et al., 2017; Jacquelin et al., 2017; Nguyen et al., 2017; Petrone et al., 2018) However, as we approach the exascale computing era, the emergence of more heterogeneous computing architectures renders non-trivial the direct application of existing algorithms and code bases to target these complex architectures. As such, for KS-DFT to remain relevant in the age of exascale and post-exascale computing, methods developers must be prepared to embrace these emerging architectures to maintain the high standard of computational performance which has come to be expected.

In recent years, the trajectory of HPC has lead to an increasing reliance on the use accelerators, such as graphics processing units (GPU), to perform the majority of the floating point operations (FLOPs) on new and emerging computing resources (Kindratenko et al., 2009; Parnell et al., 2019). For a detailed treatise on the details and challenges presented by these and other emerging architectures and their use in conjunction with electronic structure calculations, we refer to the work of Gordon et al. (2020). In this work, we limit our discussion to the optimization of KS-DFT methods on NVIDIA GPUs (in particular the NVIDIA Tesla V100) using the Compute Unified Device Architecture (CUDA) programming platform (Cook, 2012).

Recently, there has been significant research effort afforded to porting electronic structure software to the GPU (Gordon et al., 2020). In the case of large-scale calculations, much work has gone into the development of massively parallel GPU implementations of methods based on plane wave (Maintz et al., 2011; Wang et al., 2011; Jia et al., 2019), real space (Andrade and Aspuru-Guzik, 2013; Hakala et al., 2013), finite element (Das et al., 2019; Motamarri et al., 2020), and various other discretizations (Genovese et al., 2009; van Schoot and Visscher, 2016; Yoshikawa et al., 2019; Huhn et al., 2020) of the Kohn–Sham equations. In this work, we consider the Gaussian basis set discretization of the Kohn–Sham equations (Pople et al., 1992), which poses a number of challenges for GPU implementations. The majority of these challenges revolve around the computation of molecular integrals over Gaussian basis functions. Of the required integrals, the electron repulsion integrals (ERIs) and the exchange-correlation (XC) potential are among the most costly and the most challenging to port to GPU architectures. Over the years, there has been a considerable amount of research devoted to porting implementations of Gaussian basis set KS-DFT to the GPU (Yasuda, 2008; Brown et al., 2010; Titov et al., 2013; Luehr et al., 2016; Kussmann and Ochsenfeld, 2017; Manathunga et al., 2020; Peters et al., 2020); however, the vast majority of this work has been centered around the evaluation and digestion of the ERIs in the construction of the Fock matrix (Ufimtsev and Martinez, 2008, 2009a,b; Asadchev et al., 2010; Miao and Merz, 2013; Kalinowski et al., 2017; Kussmann and Ochsenfeld, 2017; Laqua et al., 2020). On the other hand, the XC potential has received much less treatment in the literature in this regard (Yasuda, 2008; Luehr et al., 2016; Manathunga et al., 2020). This disparity is understandable due to the fact that for large systems, the ERI-related contributions to the Fock matrix are computationally dominant and the most challenging to parallelize. However, with recent advances in semi-numerical techniques for exact exchange, which have shown great promise in early GPU implementations (Laqua et al., 2020), ERI-dominated calculations are quickly becoming computationally competitive with the evaluation of the XC potential by current methods. Further, current accounts of GPU implementations of the XC integration have been limited to the devices which are accessible within a particular compute node. To the best of the authors' knowledge, there does not exist a GPU accelerated distributed memory evaluation of the XC potential using Gaussian basis sets as of this report. Thus, in this work, we propose a three-level parallelism scheme for the scalable distributed evaluation of the Gaussian basis XC potential on large clusters of GPUs.

In general, there are a number of important features of GPU architectures one must consider in the development of high-performance software:

- GPU architectures exhibit orders of magnitude more computational threads than CPU architectures, allowing for the expression of massive concurrency within a single GPU device.
- The memory space which is directly accessible to GPU devices is much lower in capacity in comparison with their CPU counterparts (O(16–32 GB) on the GPU in comparison to upwards of O(1 TB) on the CPU).
- Memory access within device memory exhibits a much higher bandwidth than CPU memory (O(900 GB/s) on the GPU in comparison to O(20–50 GB/s) on the CPU).
- Data transfers between host and device memory spaces are low bandwidth [O(80 GB/s) with advanced technologies such as NVLink, O(35 GB/s) over PCIe], thus data transfers often pose a non-trivial overhead in GPU applications which require movement of large volumes of data.

A consequence of these features is that, despite the large number of threads that are available to the GPU to perform computation, data locality must be carefully tuned to exploit the low capacity device memory as to allow for the expression of concurrency but also to avoid high cost and inherently serial data transfers between host and device. As such, those algorithms which are able to express massive concurrency on local data without being interrupted by synchronization points such as data transfers and memory allocations are typically the best suited for GPU application. A key aspect of the method proposed in this report is the optimization of data movement within the XC integration as to express massive concurrency using data that resides in device memory without transfers between host and device.

Scientific applications often rely on the existence of highly tuned linear algebra libraries (such as vendor implementations of BLAS and LAPACK) to achieve high levels of performance on contemporary and emerging architectures (Dongarra et al., 1998). Over the years, many areas of matrix computation have achieved significant performance improvements through the use of GPU accelerators (Fatahalian et al., 2004; Kurzak et al., 2012; Herault et al., 2019). However, unless the matrix computations needed by a particular application are large enough as to fully exploit the resources of the device, it is unlikely that single matrix operation such as matrix–matrix multiplication will be able to achieve high computational occupancy on the device. An important achievement in high-performance numerical linear algebra has been the advent of highly tuned batched implementations of commonly encountered matrix operations, such as matrix–matrix multiplication, triangular factorization, etc. (Haidar et al., 2015; Abdelfattah et al., 2016a). Such batched implementations are provided in both vendor tuned (such as cuBLAS and cuSOLVER provided by NVIDIA) and open source (such as MAGMA, Nath et al., 2010; Tomov et al., 2010; Abdelfattah et al., 2016b) GPU accelerated linear algebra libraries. In these batched implementations, efficiency is achieved by dramatically increasing the throughput of the matrix operations via concurrent execution within a single device. Thus, if an application requires the manipulation of many small matrices in a manner that allows for concurrent execution (such as KS-DFT), large performance improvements can be made by utilizing these batched implementations (see e.g., Das et al., 2019). GPU-accelerated BLAS has previously been used in the context of XC computations (Yasuda, 2008). In this work, we examine the use of batched BLAS to further accelerate these operations to improve overall time-to-solution.

This work will be organized as follows. Sections 2.1 and 2.2 will briefly review the pertinent theory and high-level algorithmic constructs related to the XC integration. Section 2.3 will then describe the proposed method for the scalable, three-level parallelism scheme for the distributed XC integration on clusters of GPUs. Section 3 will demonstrate the performance and scalability of the purposed method in comparison to an existing high-performance CPU implementation using a wide range of molecules, basis sets, and quadrature sizes. Finally, section 4 will conclude this work and offer insight into the impact of the purposed method and briefly discuss future research directions.

## 2. Methods

### 2.1. Kohn–Sham Density Functional Theory

In KS-DFT, the total electronic energy within a particular density functional approximation (DFA) takes the form (Parr and Yang, 1994)

(1) $$\begin{array}{l} E^{tot}=T_{s}+V_{ne}+J-c_{x}K+E^{xc}, \end{array}$$

where $T_{s}$ and $V_{ne}$ are the (non-interacting) kinetic and electron-nuclear attraction energies, and J and K are the classical Coulomb and exact exchange energies, respectively. *c*_*x*_ ∈ ℝ is a parameter that scales the contribution of exact-exchange to the electronic energy. *c*_*x*_ = 0 is used for “pure” DFAs, whereas DFAs that use *c*_*x*_ ≠ 0 are referred to as “hybrid” DFAs (Becke, 1993). Without loss of generality in the following, we will take *c*_*x*_ = 0, though we note that the algorithms presented in the following sections may also be extended to hybrid methods without modification. $E^{xc}$ is the exchange-correlation (XC) energy which is taken to be a functional of the electron density ρ:ℝ^3^ → ℝ. In this work, we restrict our discussion to spin-restricted DFAs within the generalized gradient approximation (GGA) (Perdew, 1986; Perdew and Yue, 1986), i.e. $E^{xc}$ is approximated to only depend on ρ and its gradient ∇ρ:ℝ^3^ → ℝ^3^. We note for completeness that the information presented in this and the following sections may be extended to both spin-unrestricted and spin-generalized KS-DFT methods as well as more advanced DFAs (such as the meta-GGA) with the addition of only a few intermediates (Egidi et al., 2017; Petrone et al., 2018). As ∇ρ is a vector valued quantity, and thus dependent on reference frame quantities such as molecular orientation, it is canonical to express $E^{xc}$ as

(2) $$\begin{array}{l} E^{xc}=\int_{{\mathbb{R}}^{3}}\varepsilon \left(\left\{U\left(\text{r}\right)\right\}\right)\rho \left(\text{r}\right){\text{d}}^{3}\text{r}, \end{array}$$

where ε is an energy density that depends on a set of so-called “U” -variables, {*U*(**r**)}, which are independent of reference frame. Within the GGA, the canonical choice for these variables are {*U*(**r**)} = {ρ(**r**), γ(**r**)} with γ(**r**) = ||∇ρ(**r**)||.

By expanding the density in a finite set of basis functions, $S={\left\{\phi_{\mu }\left(\text{r}\right)\right\}}_{\mu =1}^{N_{b}}$,

(3) $$\begin{array}{l} \rho \left(\text{r}\right)=\underset{\mu \nu }{\sum }P_{\mu \nu }\phi_{\mu }\left(\text{r}\right)\phi_{\mu }\left(\text{r}\right), \end{array}$$

where **P** is the density matrix, the Kohn–Sham Fock matrix takes the form (Parr and Yang, 1994)

(4) $$\begin{array}{l} \text{F}=\text{h}+\text{J}+{\text{V}}^{xc}. \end{array}$$

**h** is the basis representation of the density-independent core Hamiltonian (e.g., the sum of kinetic energy and external potential operators), and **J** is the basis representation of the classical Coulomb operator. Note that we have dropped the exact exchange term in Equation (1) as we have taken *c*_*x*_ = 0. **V**^*xc*^ is the XC potential that may be expressed as (Yasuda, 2008; Burow and Sierka, 2011; Petrone et al., 2018)

(5) $$\begin{array}{l} V_{\mu \nu }^{xc}=\int_{{\mathbb{R}}^{3}}\phi_{\mu }\left(\text{r}\right)Z_{\nu }\left(\text{r}\right)+Z_{\mu }\left(\text{r}\right)\phi_{\mu }\left(\text{r}\right){\text{d}}^{3}\text{r}, \end{array}$$

where

(6) $$\begin{array}{l} Z_{\mu }\left(\text{r}\right)=\frac{1}{2}\frac{\partial \varepsilon \left(\left\{U\left(\text{r}\right)\right\}\right)}{\partial \rho }\phi_{\mu }\left(\text{r}\right)+2\frac{\partial \varepsilon \left(\left\{U\left(\text{r}\right)\right\}\right)}{\partial \gamma }\nabla \rho \left(\text{r}\right)\cdot \nabla \phi_{\mu }\left(\text{r}\right). \end{array}$$

Note that the partial derivatives of ε are to be evaluated with the U-variables calculated at argument of *Z*_μ_.

Equations (4) to (6) are general to any (real-valued) basis set expansion. In this work, we consider atomically centered contracted Gaussian basis functions of the form

(7) $$\begin{array}{l} \phi_{\mu }\left(\text{r}\right)={\left(x-R_{x}\right)}^{l}{\left(y-R_{y}\right)}^{m}{\left(z-R_{z}\right)}^{n}\overset{n_{\xi }^{\mu }}{\underset{\xi =1}{\sum }}d_{\xi }^{\mu }\exp\left(-\alpha_{\xi }^{\mu }{\left(\text{r}-{\text{R}}_{\mu }\right)}^{2}\right), \end{array}$$

where **R**_μ_ = {*R*_*x*_, *R*_*y*_, *R*_*z*_}, $n_{\xi }^{\mu }$ is the contraction depth, $d_{\xi }^{\mu }$ is a contraction coefficient, and *L* = *l* + *m* + *n* is the total angular momentum. Each term in the sum is referred to as a primitive Gaussian function. Contracted basis functions with the same *L*, $\left\{d_{\xi }^{\mu }\right\}$, $\left\{\alpha_{\xi }^{\mu }\right\}$, and **R**_μ_ will be referred to as a basis shell. Functions of the form Equation (7) are referred to as Cartesian Gaussian functions, and each Cartesian shell with angular momentum *L* consists of *L*(*L* + 1) functions. For *L* > 1, there is often a linear dependency among the functions within each Cartesian shell, which may be addressed by transforming these shells to a set of spherical Gaussian functions (Schlegel and Frisch, 1995). Each spherical Gaussian shell consists of 2*L* + 1 linearly independent functions. Not all Gaussian basis sets that consist of functions with *L* > 1 require this transformation to be linearly independent, and we will note when such a transformation has taken place.

### 2.2. Numerical Integration of Molecular Integrands

Even for the simplest forms of ε, neither Equation (2) nor Equation (5) admits analytic expressions, thus these integrations must be performed numerically. For molecular integrands, i.e., integrands with non-trivial behavior in the vicinity of atomic nuclei in polyatomic systems, a particularly attractive approach is to perform the numerical integration as a sum over weighted atomic integrands (Becke, 1988). For a molecular integrand *f*:ℝ^3^ → ℝ, we may decompose its integral over ℝ^3^ as

(8) $$\begin{array}{l} \int_{{\mathbb{R}}^{3}}f\left(\text{r}\right){\text{d}}^{3}\text{r}=\overset{N_{A}}{\underset{A=1}{\sum }}I_{A}\left[f\right],\;I_{A}\left[f\right]=\int_{{\mathbb{R}}^{3}}p_{A}\left(\text{r}\right)f\left(\text{r}\right)\text{d}b^{3}\text{r}, \end{array}$$

where *N*_*A*_ is the number of atoms, and $p_{A}:{\mathbb{R}}^{3}\to \mathbb{R}$ is an atomic partition function that obeys $\sum_{A}p_{A}\left(\text{r}\right)=1$, ∀**r** ∈ ℝ^3^. Each atomic integrand *I*_*A*_[*f*] may then be approximated by a quadrature rule

(9) $$\begin{array}{l} I_{A}\left[f\right]\approx \underset{i\in Q_{A}}{\sum }w_{i}^{A}f\left({\text{r}}_{i}^{A}\right),\;w_{i}^{A}=p^{A}\left({\text{r}}_{i}^{A}\right)w_{i}^{q} \end{array}$$

where $Q_{A}={\left\{\left(w_{i}^{A},{\text{r}}_{i}^{A}\right)\right\}}_{i=1}^{N_{g}^{A}}$ is a set of quadrature points indexed by *i* centered around the *A*-th nucleus with atomically scaled quadrature weights $w_{i}^{A}$. $\left\{w_{i}^{q}\right\}$ is the set of unmodified weights associated with the base quadrature around a particular nucleus. For convenience in the following, we define the total quadrature

$$\begin{array}{l} Q=\underset{A}{⋃}Q_{A}={\left\{\left(w_{i},{\text{r}}_{i}\right)\right\}}_{i=1}^{N_{g}}, \end{array}$$

where $N_{g}=\sum_{A}N_{g}^{A}$ is the total number of grid points needed to perform the numerical integration over the molecular integrand. Note that *w*_*i*_ is assumed to have the proper atomic scaling per Equation (9).

There are many possible choices for both the atomic partitioning scheme (Becke, 1988; Stratmann et al., 1996; Laqua et al., 2018; Aprà et al., 2020) and base quadratures around each atomic center (Becke, 1988; Murray et al., 1993; Treutler and Ahlrichs, 1995; Mura and Knowles, 1996; Gill and Chien, 2003; Aprà et al., 2020). In this work, we will use the following:

- For the atomic partition function, we will use the scheme proposed by of Stratmann, Scuseria, and Frisch (SSF) (Stratmann et al., 1996).
- For the base atomic quadrature, we will use a spherical product grid consisting of the Mura-Knowles (MK) quadrature (Mura and Knowles, 1996) for the radial integration and the Lebedev–Laikov quadrature (Lebedev, 1976) for the angular integration.

These schemes are chosen in part for the simplicity and robustness, as well as their standard use in industry KS-DFT software. Further, while it is standard practice to perform angular grid pruning to reduce the number of grid points in these product quadratures (Gill et al., 1993; Chien and Gill, 2006; Laqua et al., 2018), we perform no such procedure here. We note that the methodological details presented in this work are largely independent of such choices.

**Figure 1:**
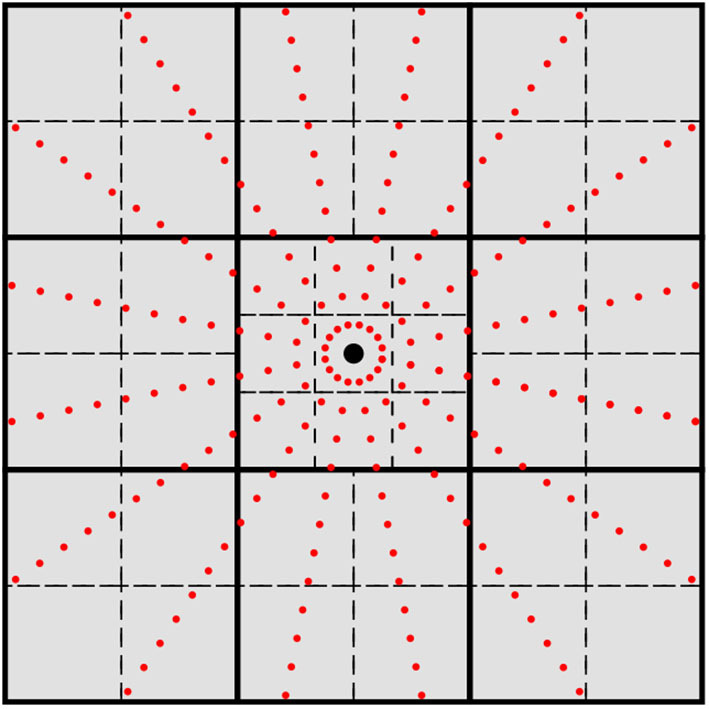
2-D cross-section of the grid batching scheme used in this work. The large black dot represents an atomic center and the small red dots represent quadrature points for spherical integration. Thick solid lines represent the initial cuboid partition, and dashed lines represent the next partition level. Atomic centered cuboids are partitioned into 27 cubical domains while off-center cuboids are partitioned into octants.

It is well-known that a naive application of Equations (8) and (9) to evaluate **V**^*xc*^ and $E^{xc}$ is very inefficient (Stratmann et al., 1996). This is due to the fact that while Gaussian functions of the form Equation (7) do not admit compact support, their exponential character yields numerically negligible contributions when evaluated far from their center. As such, Gaussians of this form may be approximated to have compact support on a sphere centered at their **R**_μ_ with cutoff radius (Burow and Sierka, 2011)

(10) $$\begin{array}{l} r_{\mu }^{cut}=\underset{\xi }{\max}\sqrt{\frac{1}{\alpha_{\xi }^{\mu }}\left(\frac{\ln\alpha_{\xi }^{\mu }}{2}-\ln\;\eta \right)}, \end{array}$$

where η is a tolerance for which |ϕ_μ_| < η for all points outside of the sphere. In this work, we have chosen η = 10^−10^. Remark that the cutoff radius only depends on the exponential coefficients, and thus may be calculated at the level of basis shell rather than individual functions for *L* > 0. Given this cutoff criteria, one may form a list of basis shells that are non-negligible for each quadrature point. Rather than check each individual quadrature points against *r*^*cut*^ for each basis shell's cutoff radius, it is canonical to group quadrature points that are spatially close into batches and perform the coarse-grained screening for non-negligible basis shells at the batch level rather than the quadrature points themselves. This procedure is known as micro-batching (Stratmann et al., 1996) and is one of the primary mechanisms by which linear scaling (with respect to system size) is achieved in the evaluation of the XC potential. Given quadrature micro-batches with a sufficiently small spatial extent, basis screening via Equation (10) produces an approximately constant number of basis functions per quadrature batch, thus leading to an overall scaling that depends only on the number of quadrature points. There are several ways to obtain the quadrature batches (Stratmann et al., 1996; Burow and Sierka, 2011; Manathunga et al., 2020). In this work, we recursively subdivide the domain spanned by the quadrature points into cuboids until the number of quadrature points within each cuboid is below a certain threshold. In this work, we have chosen this threshold to be 512 quadrature points. In practice, this partitioning scheme produces batches similar to the octree method of Manathunga et al. (2020). However, rather than bisecting every domain into octants, cuboids that contain an atomic center are partitioned into 27 cuboids as shown in Figure 1. Our experiments show that this procedure produces fewer batches with the same non-negligible shell list, which in turn improves the performance of the load balancing scheme discussed later in this section. However, much like the choice of atomic quadrature and partition functions, the choice of batching scheme does not affect the methodological details presented in this work just as long as the batches produced are able to produce sufficiently short lists of non-negligible basis shells. For a total quadrature Q, we denote the set of quadrature batches produced by this procedure as $B=\left\{B_{j}\right\}$ such that

(11) $$\begin{array}{l} Q=\underset{B_{j}\in B}{⋃}B_{j},\;\text{s.t.}\;B_{j}\cap B_{k}=\emptyset ,\text{ for }j\neq k. \end{array}$$

In the case where the batches are defined by non-overlapping cuboids surrounding an atomic center, basis shell screening may be accomplished by calculating the point of closest approach between the cuboid defining the batch and the spheres defined by center **R**_μ_ and radius $r_{\mu }^{cut}$ (Arvo, 2013). A description of this procedure is given in Algorithm 1. For $B_{j}\in B$, we define the list of non-negligible basis functions for $B_{j}$ as $S_{j}$, the number of non-negligible basis functions as $N_{b}^{j}=|S_{j}|$, and the number of quadrature points in the batch as $N_{g}^{j}=|B_{j}|$.

**Algorithm 1:**
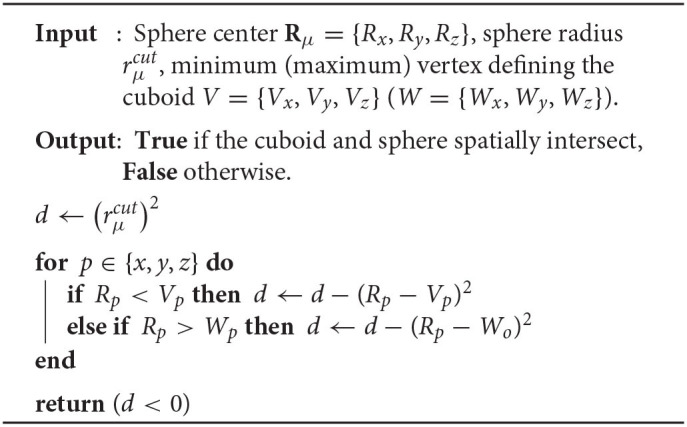
Basis shell screening via cuboid–sphere intersection.

Another advantage of quadrature batching is the ability to cast the evaluation of **V**^*xc*^ and $E^{xc}$ in terms of efficient level-1 BLAS operations such as dot products (DOT) and level-3 BLAS operations such as matrix–matrix multiplication (GEMM) and symmetric rank-2K updates (SYR2K). For a particular batch $B_{j}$, we may define a batch collocation matrix (**Φ**^*j*^) and a local density matrix (**P**^*j*^) as

(12a) $$\begin{array}{l} \Phi_{\mu i}^{j}=\{\begin{array}{ll} \phi_{\mu }\left({\text{r}}_{i}\right), & \text{for }i\in B_{j}\text{ and }\mu \in S_{j}\\ 0, & \text{otherwise.} \end{array} \end{array}$$

(12b) $$\begin{array}{l} P_{\mu \nu }^{j}=\{\begin{array}{ll} P_{\mu \nu }, & \text{for }\mu ,\nu \in S_{j}\\ 0, & \text{otherwise.} \end{array} \end{array}$$

In the following, we will refer to the extent to which **Φ**^*j*^ and **P**^*j*^ are numerically zero due to basis function screening as their local sparsity. This yields the following expressions for the density and its gradient evaluated on the quadrature points within $B_{j}$,

(13) $$\begin{array}{l} \rho_{i}^{j}=\underset{\mu \in S_{j}}{\sum }\Phi_{\mu i}^{j}X_{\mu i}^{j},\;\text{(DOT)} \end{array}$$

(14) $$\begin{array}{l} \nabla \rho_{i}^{j}=2\underset{\mu \in S_{j}}{\sum }\nabla \Phi_{\mu i}^{j}X_{\mu i}^{j},\;\text{(DOT)} \end{array}$$

(15) $$\begin{array}{l} {\text{X}}^{j}={\text{P}}^{j}\Phi^{j}.\;\text{(GEMM)} \end{array}$$

It should be understood from the context that the free index *i* is restricted to quadrature points in $B_{j}$. Given these expressions, we may now express the XC-related quantities as (Petrone et al., 2018)

(16) $$\begin{array}{l} E^{xc}=\underset{B_{j}\in B}{\sum }\underset{i\in B_{j}}{\sum }\varepsilon_{i}^{j}\rho_{i}^{j},\;\text{(DOT)} \end{array}$$

(17) $$\begin{array}{l} V_{\mu \nu }^{xc}=\underset{B_{j}\in B}{\sum }V_{\mu \nu }^{j}, \end{array}$$

(18) $$\begin{array}{l} {\text{V}}^{j}={\text{Z}}^{j}\Phi^{j,T}+\Phi^{j}{\text{Z}}^{j,T},\;\text{(SYR2K)} \end{array}$$

with

(19) $$\begin{array}{l} \varepsilon_{i}^{j}=w_{i}\varepsilon \left(\left\{U\left({\text{r}}_{i}\right)\right\}\right),\;\frac{\partial \varepsilon_{i}^{j}}{\partial \rho }=w_{i}\frac{\partial \varepsilon \left(\left\{U\left({\text{r}}_{i}\right)\right\}\right)}{\partial \rho }\\ \frac{\partial \varepsilon_{i}^{j}}{\partial \gamma }=w_{i}\frac{\partial \varepsilon \left(\left\{U\left({\text{r}}_{i}\right)\right\}\right)}{\partial \gamma }, \end{array}$$

(20) $$\begin{array}{l} Z_{\mu i}^{j}=\frac{1}{2}\frac{\partial \varepsilon_{i}^{j}}{\partial \rho }\Phi_{\mu i}^{j}+2\frac{\partial \varepsilon_{i}^{j}}{\partial \gamma }\left(\nabla \rho_{i}^{j}\cdot \nabla \Phi_{\mu i}^{j}\right). \end{array}$$

For brevity in the following, we define for $i\in B_{j}$

(21) $$\begin{array}{l} \rho^{j}=\left\{\rho_{i}^{j}\right\},\;\nabla \rho^{j}=\left\{\nabla \rho_{i}^{j}\right\},\;\varepsilon^{j}=\left\{\varepsilon_{i}^{j}\right\},\\ \varepsilon_{\rho }^{j}=\left\{\frac{\partial \varepsilon_{i}^{j}}{\partial \rho }\right\},\;\varepsilon_{\gamma }^{j}=\left\{\frac{\partial \varepsilon_{i}^{j}}{\partial \gamma }\right\}. \end{array}$$

**Figure 2:**
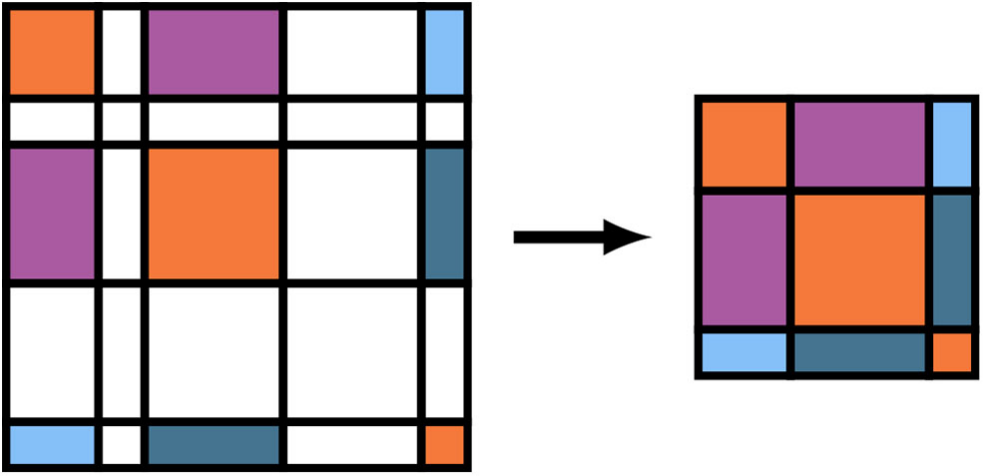
Batch matrix compression scheme for operator basis representations relative to non-negligible function indices. Colored tiles represent matrix elements that are to be included in the compressed matrix, and white tiles represent matrix elements that are to be neglected. Note that these do not necessarily correspond to zeros/non-zeros in the original matrix.

As written, the GEMM and SYR2K given in Equations (15) and (18) are block sparse level-3 BLAS operations, i.e. BLAS operations involving matrices which contain many blocks which are numerically zero. To avoid performing unnecessary FLOPs in the evaluation of these intermediates, it is possible to store the batch local matrices in Equations (12b), (15), and (18) in a compressed format which stores the blocks corresponding to non-negligible basis shells contiguously and explicitly removes the zeros from related computation (Stratmann et al., 1996). A pictorial representation of this matrix compression for the density matrix is given in Figure 2. We note for completeness that the forms of Equations (15) and (18) do not change under this compression, but the sizes of the free indices (as well as the contracted index in the case of Equation 15) are reduced. To avoid a full decompression of the batched **V**^*j*^ intermediates, Equation (17) may be implemented by simply incrementing the blocks of the full dimensional **V**^*xc*^ by the corresponding blocks of **V**^*j*^ for each *j*. Note that compression of **Φ**^*j*^, **X**^*j*^, and **Z**^*j*^ need not be explicit in that they may be evaluated directly in compressed form.

### 2.3. Distributed Parallel Implementation on Clusters of GPU Accelerators

In this section, we propose a three-level parallelism scheme for the distributed evaluation of **V**^*xc*^ and $E^{xc}$. A schematic representation of this procedure is given in Algorithm 2. For simplicity in the following discussion, we will assume MPI message passing for distributed computation. Parallelism will be expressed at the following levels:

1. Concurrent evaluation of the quadrature batches between independent computing ranks;
2. Concurrent evaluation of the quadrature batches assigned to a particular computing rank;
3. Concurrency within the evaluation of a particular quadrature batch to evaluate terms such as the atomically scaled quadrature weights, batch collocation and local density matrices, the level-3 BLAS operations of Equations (15) and (18), etc.

In the context of the batching scheme discussed in section 2.2, ensuring proper local sparsity in the batch local **P**^*j*^ and **Φ**^*j*^ typically generates a large number of relatively small batches that must be evaluated. As the work required to evaluate a single $B_{j}$ is typically small, distributing its evaluation would be inefficient. Given that **P** and **V**^*xc*^ can be replicated in the memory spaces accessible to each the compute rank, the evaluation of each quadrature batch requires no communication. Thus, the fully distributed numerical integration of the XC quantities may be performed with only a single distributed reduction (MPI_Reduce or MPI_Allreduce) following completely independent local computation. We note for posterity that this replication need not constitute a unique copy of these matrices for each compute rank, only that these matrices are accessible from each rank, e.g. in the case of partitioned global address space (PGAS) distributed memory models such as the one provided by the GlobalArrays library, it would be sufficient to keep a single copy of these matrices within the memory accessible to a single compute node. However, in this work, we do not explore the use of PGAS memory models, thus the replication will be performed at the rank level.

**Algorithm 2:**
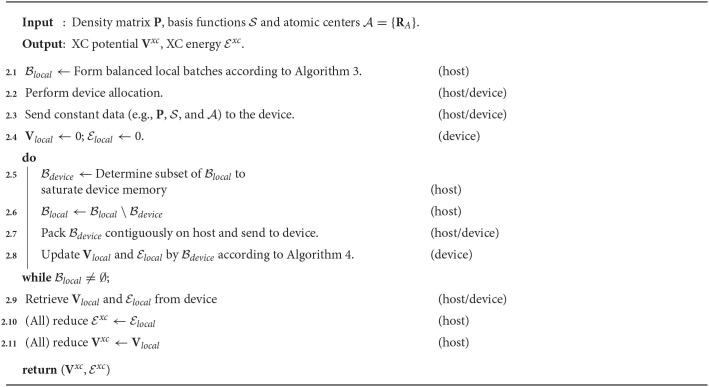
Parallelism scheme for the evaluation of the XC potential and XC energy.

#### 2.3.1. Distributed Load Balance in the XC Integration

Despite this embarrassingly parallel integration procedure, care must be taken to ensure load balance among the independent ranks as the variance in the computational work required between different batches is often quite large due to differences in local sparsity and batch sizes. The simplest choice to distribute this work would be to distribute the batches at the atomic quadrature level, i.e. each rank receives the quadrature batches generated from a particular atomic quadrature. However, this scheme can lead to load imbalance as the local sparsity of the atoms far from the center of mass can often be much larger than those that are surrounded by other atoms. In this work, we choose to distribute the work at the individual batch level by approximating the FLOPs incurred by each batch,

(22) $$\begin{array}{l} W_{j}=N_{g}^{j}\left(N_{A}^{2}+9N_{b}^{j}+2{\left(N_{b}^{j}\right)}^{2}+3\right)+{\left(N_{b}^{j}\right)}^{2}. \end{array}$$

Each term in Equation (22) accounts for a rough estimate of the number of operations (FLOPs or otherwise) required for specific algorithmic kernels in the digestion of $B_{j}$ for the XC integration. The first four terms accounts for (1) the atomic weight partitioning, (2) Equations 13, 14, and 20 and the collocation matrix (and its gradient), (3) the level-3 BLAS operations in Equations 15 and 18, and (4) Equations 16 and 19. The final term in Equation (22) accounts for the packing of Equation (12b) and the increment of Equation (17). Note that *W*_*j*_ does not represent the true number of FLOPs required to evaluate intermediates associated with $B_{j}$, e.g., we do not consider FLOP estimates for evaluation of the exponential in Equation (7), nor screening in the evaluation of the atomic weight scaling, etc. However, *W*_*j*_ has empirically sufficed to produce balanced distributed computation for all problems considered. A schematic for the load balance scheme used in this work is given in Algorithm 3. There are two important remarks that should be understood from Algorithm 3. The first is that it requires no communication between independent ranks, i.e., the load balance is replicated on each processor. The second is that once the set of local batches $B_{local}$ has been determined for each processor, batches with the same $S_{j}$ are merged into a single batch (Line 3.11). The rationale behind this step is to avoid polluting the device memory with redundant copies of **P**^*j*^ and **V**^*j*^.

**Algorithm 3:**
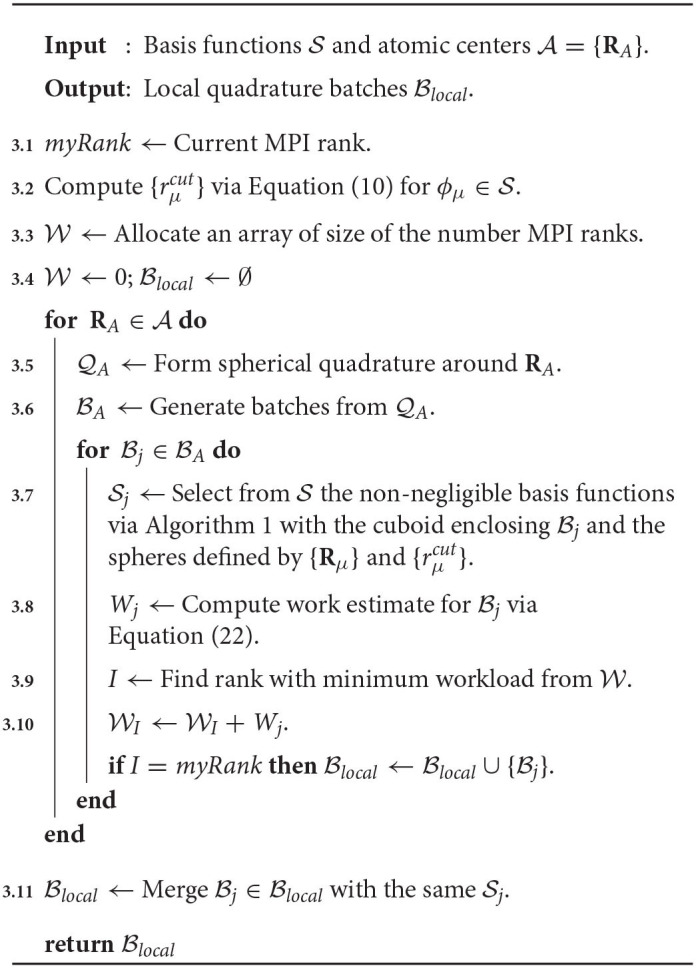
Quadrature batch load balance for distributed XC integration.

While Algorithm 3 could be implemented on the GPU, as has been discussed in the context of batch generation in related work (Manathunga et al., 2020), we do not explore such implementations in this work. To improve the performance of the CPU implementation of Algorithm 3, the loop around the atomic quadrature batches may be parallelized using shared memory parallelism schemes such as OpenMP. Further, as has been suggested by others (Yasuda, 2008), the cost of grid generation may be amortized in calculations involving many Fock matrix formations with the same nuclear geometry by forming it once for the formation of the first Fock matrix and reusing it for subsequent formations. As will be demonstrated in section 3, Algorithm 3 only becomes a computational bottleneck in the strong scaling limit for medium-to-large molecular systems.

#### 2.3.2. Local XC Integration on the GPU

Up to this point, the discussed work distribution scheme has been largely independent of whether or not the evaluation of local quadrature batches is to be performed on the host or the device. In this work, we only consider the case where a single MPI rank is driving a single device (one-to-one), i.e. we do not consider device affinities of multiple MPI ranks driving a single device (many-to-one) nor a single MPI rank driving multiple devices (one-to-many). The method proposed could be extended to one-to-many device affinities through an additional invocation of Algorithm 3 to produce balanced quadrature batches which are to be executed on a particular device. However, in the strong scaling limit, it would be unlikely that this affinity would be resource efficient due to a decrease in work assigned to any particular compute rank.

##### 2.3.2.1. Architecture of NVIDIA Tesla V100

The GPU targeted in this work is the NVIDIA Tesla V100-SXM2 using the CUDA programming environment. However, the methodological developments described in this work may be extended to any GPU device given a software stack which provides batched BLAS functionality. The V100 is equipped with 16 GB high-bandwidth global memory and 80 streaming multiprocessors (SM). Within the CUDA model, independent tasks are launched in the form of kernels and concurrency on the device is expressed in a four-level parallelism scheme:

- At the lowest level is the GPU thread that executes instructions issued by the SM.
- In contrast to CPU architectures, where all threads may execute more or less independently, the overhead of instruction issuance is mitigated on GPU devices in part by issuing a single instruction to multiple threads which execute in lock step. This is known as single-instruction multiple thread (SIMT) concurrency, and the collection of threads which execute in this manner is known as a *warp* in the CUDA vernacular. On the V100, a warp consists of 32 threads.
- Warps are then collected into groups called thread blocks, which may share data and be mutually synchronized. Thread blocks are typically comprised of 256–1024 threads which execute independently at the warp level.
- Thread blocks are further grouped into process grids which are specified at the time that the kernel is launched. A kernel has completed once all the thread blocks in its specified process grid have finished executing.

For a kernel launched with a particular process grid, thread blocks are scheduled and executed concurrently among the different SMs. Ordering of kernel execution on CUDA devices is achieved by a software construct known as a stream: kernels launched on the same stream are guaranteed to be executed in the order with which they were specified. For kernels which are designed not to achieve full occupancy within the SM, it is possible to overlap independent kernel invocations on separate streams. In this work, however, the kernels developed are designed to achieve high occupancy within each SM, thus the potential for overlap of independent kernels is minimal. Another consideration one must account for within the SIMT execution model is the concept of warp divergence, i.e. kernels that execute different instructions within a particular warp. Due to the SIMT execution model, instructions must be executed at the warp level, thus if branch logic causes the warp to diverge into *N* unique instructions, the execution time of this kernel will be roughly the sum of the execution times for the individual instructions, thus reducing the parallel efficiency of the particular kernel. Such divergence can lead to significant performance degradation. As such, one must carefully design GPU kernels such that unique instructions that are desired to execute concurrently are executed along (or near) warp boundaries to avoid such degradation.

##### 2.3.2.2. Data Locality

The algorithm presented in this work aims to maximize the potential for concurrency in the evaluation of the local quadrature batches by minimizing synchronization points, such as data transfers and memory allocations, which hinder the ability to express concurrency. As the computational work required to evaluate any particular quadrature batch is small, concurrency is achieved by batching the evaluation of the quadrature batches on the GPU. This approach has been inspired by GPU accelerated batched BLAS operations, which achieve high throughput by batching the evaluation of small matrix operations into a single kernel launch (Haidar et al., 2015; Abdelfattah et al., 2016a). Given that the data associated with a particular $B_{j}$ must reside in device memory for it to be processed (quadrature points and weights, $S_{j}$, **Φ**^*j*^, **P**^*j*^, **Z**^*j*^, etc.), the approach taken in this work is to saturate the device memory with as many quadrature batches as possible as to allow for their concurrent evaluation. Note that this approach does not change the amount of data that must be transferred between host and device throughout the XC integration, but it does reduce the frequency and improve the performance of these data transfers by saturating the bandwidth between host and device while allowing for the expression of more concurrency on the device between data transfers. In the case when all of the quadrature batches are unable to simultaneously occupy the device memory, subsets of the local quadrature batches which saturate device memory are chosen to be executed concurrently until all batches have been processed. A depiction of this procedure is given in Lines 2.5 to 2.8. The performance of these data transfers may be further improved in Line 2.7 by packing the batch data contiguously into page-locked memory (as is produced by cudaMallocHost in the CUDA SDK) on the host. In addition, rather than perform numerous memory allocations and deallocations between processing subsets of local quadrature batches, the cost of device memory allocation may be amortized by preallocating a large fraction of available device memory at the beginning of the XC integration and manually managing memory allocation throughout the calculation (Line 2.2). Note that a vast majority of the data associated with a particular $B_{j}$ need not be referenced on the host nor transferred between host and device. In essence, the only batch-specific data that need be transferred between host and device for a particular $B_{j}$ are its quadrature points and weights, the information pertaining to the atomic center which generated that batch (for the evaluation of the atomic partition function), and the information describing $S_{j}$. All other data may be allocated and manipulated directly on the device.

In addition to batch-specific data that must reside in device memory, there are a number of other quantities that are unrelated to a particular batch that are useful to store in device memory to avoid host-device transfers and to exploit the high-bandwidth memory, which is common on contemporary devices. These quantities include **P**, S, and things such as the atomic positions, inter-nuclear distances, etc. For example, in cases where **P** can reside in memory, the packing of batch local **P**^*j*^ may be made very efficient by limiting data transfers to be internal to the device memory (i.e. device memory copies). In addition, it is also advantageous to store local contributions to **V**^*xc*^ and $E^{xc}$ on the device as to avoid communication of intermediate data between the evaluation of batch subsets on the device. We note that even for the largest problem considered in this work [1,231 atoms, *N*_*b*_ = O(10,000)], both **V**^*xc*^ and **P** may reside simultaneously in device memory while leaving enough additional memory for batch-specific data as to allow for enough concurrency to be resource efficient on the device. For hypothetical problems for which this is not possible, the packing of **P**^*j*^ and the increment of **V**^*j*^ can be performed on the host at the cost of significant performance degradation. We do not explore such implementations here.

##### 2.3.2.3. Batch Execution of Quadrature Batches on the GPU

Given a set of quadrature batches that saturate device memory, Algorithm 4 depicts a general outline of the concurrency pattern for their simultaneous evaluation on a single device. Algorithm 4 exhibits a number of important features that warrant brief discussion. The first is the utilization of batched level-3 BLAS primitives for the concurrent evaluation of Equations (15) and (18) for all batches that reside in device memory (Algorithm 4). An important remark related to this batched BLAS invocation is that the batch local matrices are often not of uniform dimension for all batches in device memory. As such, they may not be implemented by uniform batched BLAS implementations, such as those provided by cuBLAS. In this work, we have used the variable-dimension batched (or “vbatched”) GEMM (VB-GEMM) and SYR2K (VB-SYR2K) implementations from the MAGMA (Nath et al., 2010; Tomov et al., 2010; Abdelfattah et al., 2016b) library to perform these batched evaluations. Another important feature of Algorithm 4 is that, while the order of operations within the various **parallel for** loops are indicative of the order with which the various tasks are executed at a high level, each of these tasks represent individual kernels for which concurrency between the separate $B_{j}$'s occurs at the thread block level. That is to say that each kernel invocation performs the **parallel for** loop as a batched invocation for each task individually. As has been discussed in similar work (Laqua et al., 2020), these operations could also be scheduled on different streams to achieve concurrency in batch execution. We do not explore such implementations in this work. Finally, much like the batched BLAS invocations, which are designed to express concurrency both within a matrix operation and between matrix operations themselves, each kernel invocation for the XC-specific tasks in Algorithm 4 is designed to express concurrency within each task as well. Each batch-local task is designed to occupy a subset of the process grid while evaluation of each batch local task is performed independently on separate subsets within the same kernel launch. In practice, this may implemented using multi-dimensional kernel launches within the CUDA framework.

**Algorithm 4:**
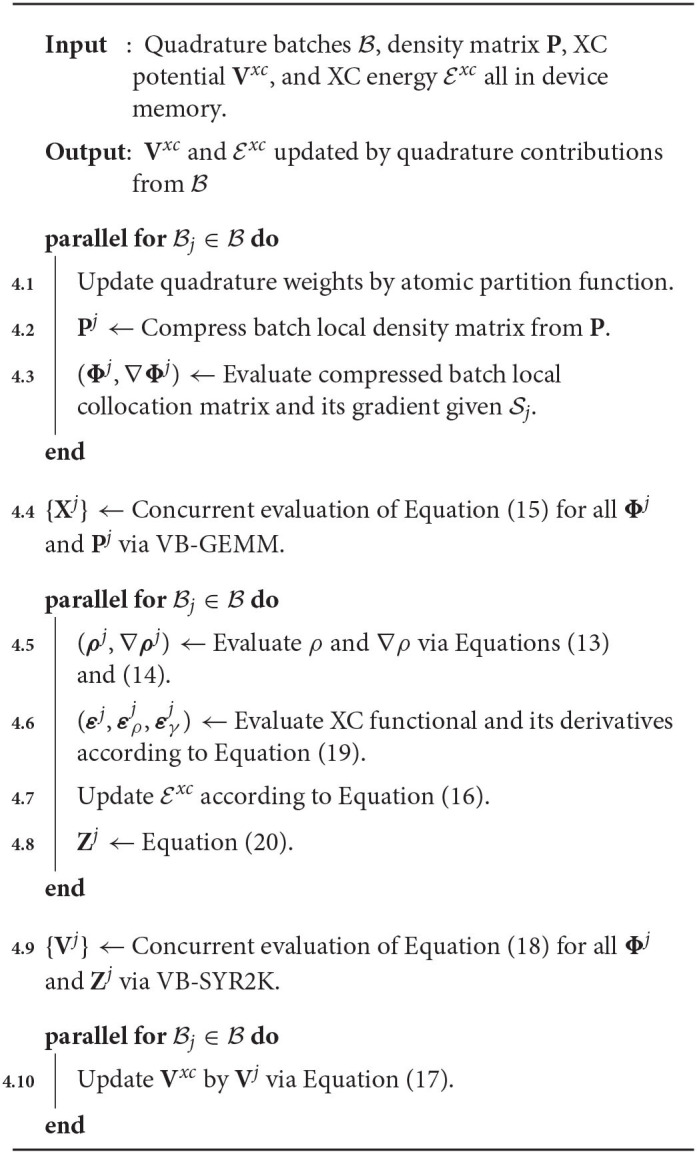
Concurrent evaluation of quadrature batches on a GPU device.

While GPU-accelerated BLAS functionality may be provided by optimized third-party libraries, as of this work there does not exist standard GPU implementations of the remainder of the operations required for the XC integration. As such, they must be implemented and optimized by hand. The details of such implementations are outside the scope of this work as they are largely dependent on the data structures used in a particular software. However, there are a few important details related to the algorithmic choices used in this work, which warrant brief discussion. In the context of the evaluation of **Φ**^*j*^ on the device, we adopt a simple strategy that assigns the evaluation of a single contracted basis shell at a particular point to a single thread, i.e., we do no express concurrency in the evaluation of the exponential factors of the primitive Gaussians. Care is taken in the implementation presented in this work to minimize the chance of warp divergence by assigning evaluations of the same basis shell at various quadrature points to the same warp (i.e., to minimize the frequency of divergence in the sum of Equation 7 with functions of differing $n_{\xi }^{\mu }$). We will demonstrate the efficacy of this simple strategy in section 3.

A major difference in the work presented here relative to existing methods for GPU XC integration (Yasuda, 2008; Manathunga et al., 2020) is the strategy for the evaluation of **ϵ**^*j*^ and its functional derivatives on the device. On the CPU, there are several standard libraries, such as Libxc (Lehtola et al., 2018) and XCFun (Ekström, 2020), which implement a vast number of XC functionals that are commonly used in KS-DFT calculations. Some work (Manathunga et al., 2020) has been dedicated to porting all or portions of these libraries to the GPU, including an initial implementation of porting Libxc to CUDA in the development version of the library itself. However, there does not exist a mature, high-performance GPU interface for these libraries at this time. To ensure the highest performance possible, the approach taken in this work has been to develop an open-source library, ExchCXX (Williams-Young, 2020), which provides the necessary functionality. ExchCXX is a modern C++ library that implements a commonly used subset of XC functionals for evaluation on the host or device though a simple, common API. We note that the numerical expressions for the XC functionals implemented in ExchCXX have been taken directly from Libxc and have been demonstrated to produce numerically indistinguishable results.

We note for posterity that, in previous work (Yasuda, 2008), the use of single precision and mixed precision arithmetic has been shown to further improve the performance of GPU-accelerated XC integration. However, as the performance gap between single and double precision arithmetic on GPU hardware has been closing in recent years (Cook, 2012), all calculations performed in this work use strictly double-precision arithmetic.

## 3. Results

In essence, the method proposed and implemented in this work (Algorithm 2) is composed of three computationally dominant phases:

1. A load balancing phase which is replicated on all MPI ranks (Algorithm 3);
2. A local integration phase which is executed on the device (Algorithm 4);
3. A reduction phase that combines the locally computed XC quantities in distributed memory to produce the final integration results.

In this section, we examine various performance characteristics of these phases as implemented in the open-source NWChemEx software package (Kowalski et al., 2020). In addition, we compare the performance and scaling of this implementation to that of an analogous scalable CPU implementation in the open-source NWChem software package (Aprà et al., 2020). We have chosen to examine the performance of the purposed method as applied to 4 molecules: Taxol, Valinomycin, Olestra, and Ubiquitin; and 2 basis sets: 6-31G(d) (Ditchfield et al., 1971; Hehre et al., 1972; Hariharan and Pople, 1973; Francl et al., 1982; Gordon et al., 1982) and cc-pVDZ (Dunning, 1989; Woon and Dunning, 1993), to provide a performance characterization for systems with a wide range of size, spacial extent, and basis dimension. The geometries and references for this structures are included in the Supplementary Material. All calculations were performed using the PBE GGA XC functional (Perdew et al., 1996). Calculations involving the 6-31G(d) basis set were performed using Cartesian Gaussian functions, while those involving cc-pVDZ were performed using spherical Gaussian functions. A list of data relevant to the performance of calculations involving these systems can be found in Table 1. In addition, we have examined the use of 3 commonly encountered atomic quadrature sizes: the fine (FG), ultra-fine (UFG), and super-fine (SFG) grids, as described in Table 2.

**Table 1:** Molecule sizes and basis dimensions.

**Molecule**	***N*_*A*_**	***N*_*b*_/6-31G(d)**	***N*_*b*_/cc-pVDZ**
Taxol	110	1,013	1,099
Valinomycin	168	1,350	1,542
Olestra	453	3,181	3,840
Ubiquitin	1,231	10,292	11,577

**Table 2:** Atomic quadrature sizes.

**Grid**	***N*_*ang*_**	***N*_*rad*_**	**$N_{g}^{A}$**
FG	302	75	22,650
UFG	590	99	58,410
SFG	974	175	170,450

All calculations have been performed on the Summit supercomputer at the Oak Ridge Leadership Computing Facility (OLCF). Each Summit node consists of 2 IBM POWER9 CPUs (2x21 @ 3.8 GHz) and 6 NVIDIA Tesla V100 GPUs. Further, the Summit supercomputer leverages an NVLINK host-device interconnect that drastically improves the bandwidth of data transfers in this work. To enable a fair comparison between NWChem and NWChemEx, each Summit node has been subdivided into 6 equally sized “resource sets” consisting of 7 CPU cores and 1 GPU. For calculations involving NWChemEx, concurrency in the CPU execution will be performed in shared memory to adhere to the one-to-one CPU-to-GPU affinity previously discussed, i.e., 1 MPI rank with 7 shared memory threads driving a single GPU. Note that CPU parallelism is only utilized in the generation of the local quadrature batches as discussed in section 2.3.1, and the launching of kernels to execute Algrithm 4 on the GPU is performed in serial.

Calculations involving NWChem were performed using a locally modified copy of release version 7.0.0. Code modifications were limited to ensuring that the radial scaling factors of the MK radial quadrature produced identical atomic quadratures to those in NWChemEx. Further, NWChem DFT calculations were performed with grid pruning disabled and using the SSF atomic partitioning scheme. Note that while the quadratures are identical between the two codes, NWChem exhibits a number of algorithmic differences with those presented in this work. These include additional density and weight screening techniques within each quadrature batch. However, these steps only improve the observed performance in NWChem, thus they do not detract from the performance comparisons made in this work. To ensure that we are comparing with consistent, replicatable performance in NWChem, all calculations have been performed using converged density matrices. Each resource set will consist of 7 MPI ranks for calculations involving NWChem as, with the exception of the atomic weight scaling, its implementation of the XC integration does not exploit shared memory parallelism. Further, we note that the use of the GlobalArrays library (Nieplocha et al., 2006; Krishnan et al., 2012) in NWChem yields that one MPI rank per physical node will be used as a progress rank for remote memory access rather than performing computation related to the XC integration.

Both NWChem and NWChemEx were compiled using the GNU 8.1.0 compiler suite (gcc, g++, gfortran) to compile host code using high levels of compiler optimization (-O3 -mcpu=native -mtune=native -ffast-math). The device code in NWChemEx was compiled using the NVIDIA CUDA compiler (nvcc) as provided in the CUDA SDK (version 10.1.105). Analogous optimization flags (-O3 --use-fast-math) as well as architecture specific flags to generate optimized binaries for CUDA compute capability 7.0 (-gencode sm_70,compute_70) were used in the compilation of device code. NWChem was linked to the serial version of the IBM Engineering Scientific Software Library (ESSL version 6.1.0) for POWER9 optimized BLAS functionality. GPU accelerated batched BLAS was provided by the MAGMA library (version 2.5.1) while non-batched BLAS for operations such as dot products was provided by the cuBLAS library from the NVIDIA CUDA SDK.

### 3.1. Integration Performance on GPU Devices

First, we examine the performance characteristics of Algorithm 2 on a single Summit node. This treatment allows us to examine the effects of molecule size, basis dimension, and quadrature size on overall GPU performance separately from scaling in a distributed setting. Strong scaling of the purposed method as well as its comparison to NWChem will be presented in the following subsection. An overall component analysis of the timings on a single Summit node is given in Table 3. The wall times presented in Table 3 are aggregated over the entire XC integration, i.e. for the local integration, the times presented are representative of the sum of all invocations that saturate device memory (*N*_*sat*_). Further, we note that these times also include the contiguous host packing and host-device transfer of batch data (i.e., all operations contained in the loop over quadrature batches in Algorithm 2). In addition, the times presented for load balancing include all operations in Algorithm 3, i.e. batch generation and the course-grained screening of basis shells at the batch level. As these calculations were performed within a single Summit node, the reduction phase is not explicitly considered in Table 3, but its contributions are included in the times labeled “Other.” As expected, although Algorithm 3 is executed on the host in this work, the dominant computational phase for these calculations was the local integration. Further, we note that the overall cost of Algorithm 3 for a particular molecule/grid pair is largely independent of basis size but scales linearly with respect to grid size for a particular molecule/basis pair. The result of this is that the relative cost of load balancing is reduced as basis size increases. However, while this cost is not dominant at low processor counts, it will be demonstrated to be dominant in the strong scaling limit in the following subsection.

**Table 3:** Aggregate wall times for computationally intensive operations of XC integration for the various problems considered.

**Molecule**	**Basis**	**Grid**	***N*_*sat*_**	**Load balance**	**(%)**	**Local integration**	**(%)**	**Other**	**(%)**	**Total**
Taxol	6-31G(d)	FG	1	0.073	(17.49)	0.310	(73.99)	0.036	(8.52)	0.419
		UFG	2	0.145	(15.50)	0.746	(79.59)	0.046	(4.91)	0.937
		SFG	3	0.252	(15.76)	1.30	(80.84)	0.055	(3.41)	1.60
	cc-pVDZ	FG	1	0.075	(14.62)	0.399	(77.59)	0.040	(7.79)	0.514
		UFG	2	0.153	(13.70)	0.918	(82.12)	0.047	(4.18)	1.12
		SFG	3	0.268	(13.26)	1.68	(83.24)	0.071	(3.50)	2.02
Valinomycin	6-31G(d)	FG	1	0.128	(14.74)	0.685	(79.14)	0.053	(6.12)	0.865
		UFG	3	0.259	(15.79)	1.33	(80.95)	0.054	(3.26)	1.64
		SFG	5	0.446	(14.98)	2.45	(82.21)	0.084	(2.81)	2.98
	cc-pVDZ	FG	2	0.136	(12.17)	0.916	(82.27)	0.062	(5.55)	1.11
		UFG	3	0.274	(11.99)	1.96	(85.74)	0.052	(2.27)	2.29
		SFG	6	0.474	(11.09)	3.70	(86.61)	0.098	(2.30)	4.27
Olestra	6-31G(d)	FG	2	0.433	(23.60)	1.20	(65.45)	0.201	(10.95)	1.84
		UFG	5	0.872	(23.48)	2.65	(71.39)	0.191	(5.13)	3.72
		SFG	9	1.49	(21.79)	5.14	(75.13)	0.211	(3.08)	6.84
	cc-pVDZ	FG	3	0.481	(19.87)	1.68	(69.48)	0.258	(10.66)	2.42
		UFG	6	0.953	(19.59)	3.63	(74.57)	0.284	(5.83)	4.87
		SFG	11	1.63	(18.54)	6.92	(78.53)	0.259	(2.94)	8.82
Ubiquitin	6-31G(d)	FG	22	3.12	(10.94)	22.5	(78.90)	2.89	(10.15)	28.5
		UFG	45	6.01	(10.84)	47.5	(85.70)	1.92	(3.46)	55.4
		SFG	84	10.2	(9.94)	90.2	(87.82)	2.30	(2.24)	103
	cc-pVDZ	FG	30	3.44	(7.83)	38.2	(86.96)	2.29	(5.21)	43.9
		UFG	61	6.64	(7.50)	79.6	(89.80)	2.40	(2.71)	88.6
		SFG	111	11.2	(7.04)	145	(90.90)	3.30	(2.07)	160

*All times are given in seconds and N_sat_ is the number of times the device memory was saturated in Algorithm 2 to complete the integration*.

In this work, we focused on two algorithmic motifs that are important for the XC integration on the GPU:

1. Optimizing data locality to minimize the overhead of low-bandwidth data transfers between host and device and to maximize the potential to express concurrency without synchronization, and
2. Batching together the evaluation of small tasks on the device through the use of kernels that express concurrency both within a quadrature batch and between batches to improve throughput on the device.

**Figure 3:**
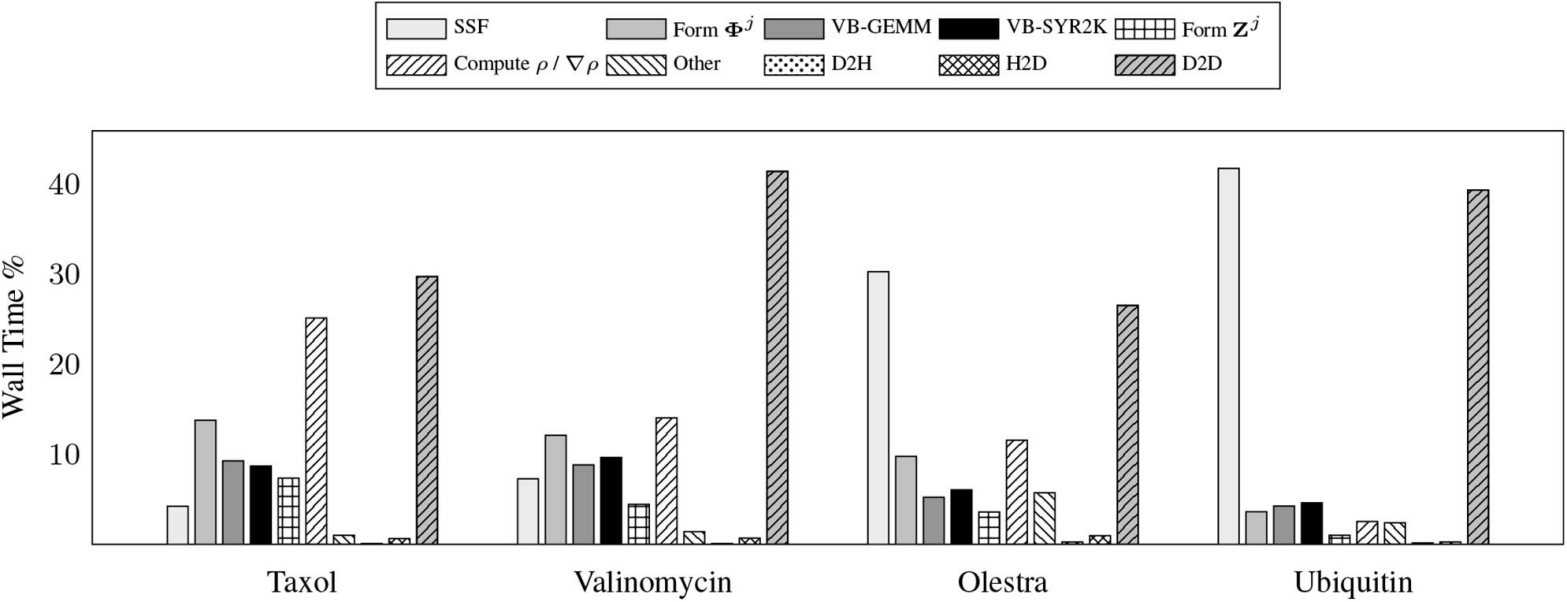
Wall time percentages for various operations in the XC integration involving the graphics processing units (GPU), which includes host-to-device (H2D), device-to-host (D2H), and device-to-device (D2D) transfers.

To demonstrate the efficacy of these motifs, we examine the relative costs of the various compute and memory intensive operations incurred by the various kernels during the local integration on the device. Due to the fact that GPU computation is generally asynchronous with respect to host computation, care must be taken in accruing accurate performance data relating to individual kernels as to not impede computational progress on the device. For this purpose, we have utilized the NVIDIA profiler nvprof to obtain kernel level performance metrics. A summary of the overall time spent on various operations involving the GPU for the UFG basis and 6-31G(d) basis set is provided in Figure 3.

There are a number of important features exemplified in the results presented in Figure 3. The first is that saturating the device memory to ensure data locality all but removes the cost of host-to-device (H2D) and device-to-host (D2H) data transfers, yielding < 1% of the overall computational cost combined for all problems considered. For the smaller test cases (Taxol and Valinomycin), the GPU implementation is dominated by the evaluation of ρ / ∇ρ and device-to-device (D2D) memory transfers. For the larger test cases (Olestra and Ubiquitin), the integration is dominated by the evaluation of the SSF atomic partition weights and D2D memory transfers. We note for clarity that the D2D transfers are intra-GPU device memory copies, not inter-GPU communication. The times for the evaluation of the XC functional on the device are not explicitly shown in Figure 3 as they are negligibly small. They are however included in the “Other” timing accumulations.

**Figure 4:**
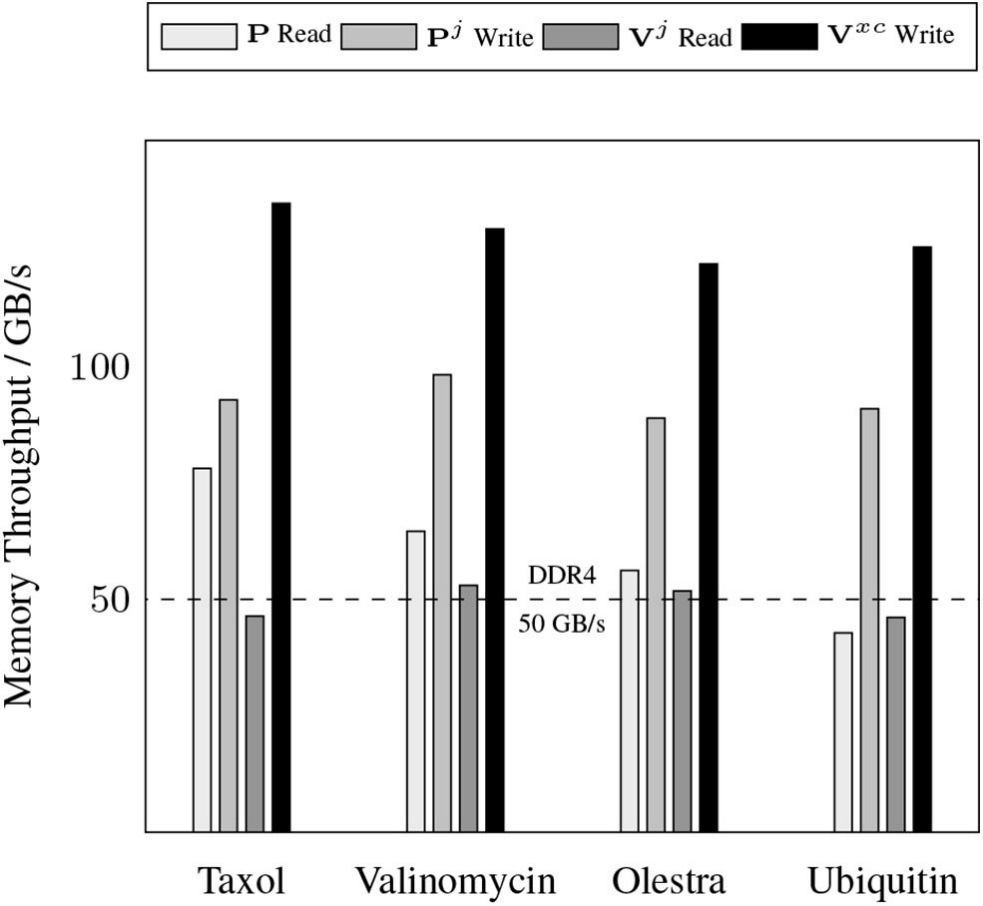
Achieved memory throughput for dominant device-to-device (D2D) data transfers in the XC integration compared to the peak DDR4 bandwidth in host memory.

A somewhat unexpected result is the dominant cost posed by intra-GPU D2D transfers for all problems considered. The D2D timings including the packing of Equation (12b), the incrementing of Equation (17), and various other small D2D transfers such as those involving storage of the basis functions. This result is unexpected due to the high-bandwidth of memory transfers within device memory. To further examine the details of this unexpected dominant cost, Figure 4 shows the achieved memory read and write throughputs for the intra-GPU data transfers incurred by the batch kernels that implement Equations (12b) and (17). These achieved throughputs are compared to the peak bandwidth of DDR4 (CPU) memory: 50 GB/s. For these kernels, we are able to achieve a memory throughput of O(100 GB/s) for data writes and between 50 and 70 GB/s for data reads, with the throughput for data reads decreasing with increasing system size. This decrease in data read throughout with system size is likely due to memory bank conflicts arising from multiple GPU threads accessing the same memory address simultaneously. Although these kernels are not able to achieve memory throughput reflective of peak device memory bandwidth (900 GB/s) due to their access of non-coalesced, non-contiguous memory, they far exceed the throughput that would be achievable in CPU memory. Further, as the memory footprint of these packed matrices are among the largest in the purposed method, exploiting intra-GPU memory transfers avoids additional H2D and D2H transfers which would pose non-trivial costs due to their low bandwidth.

**Figure 5:**
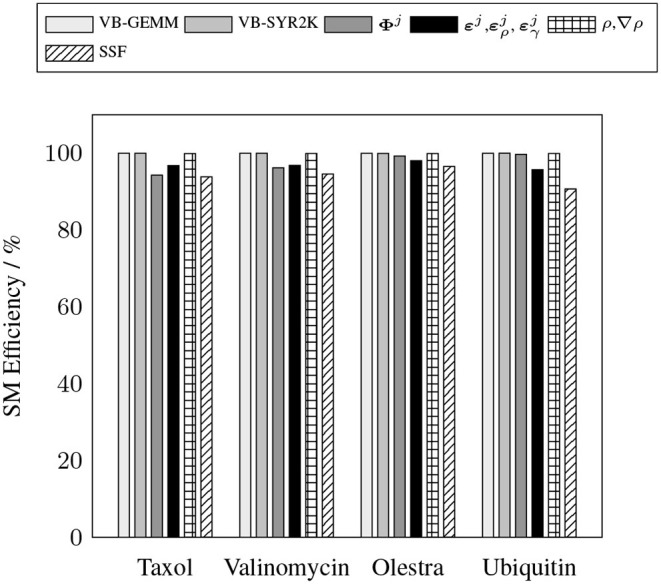
Achieved SM efficiency for batched kernels in the XC integration.

**Figure 6:**
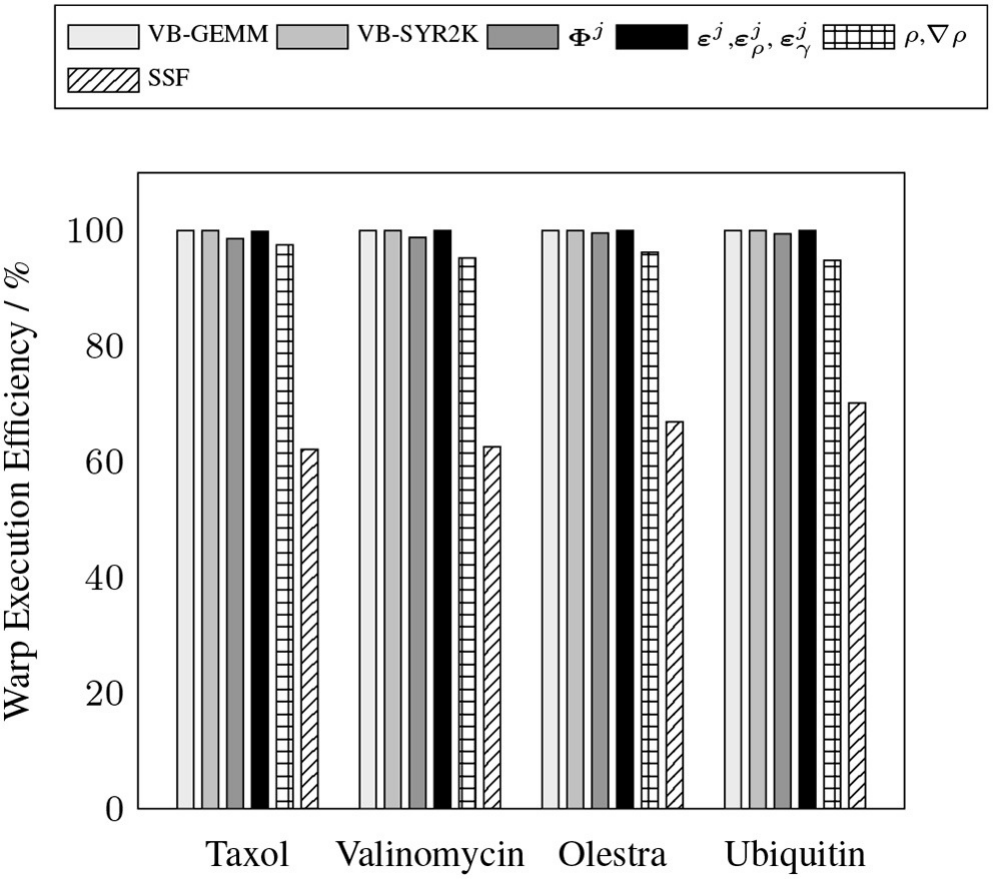
Achieved warp execution efficiency for batched kernels in the XC integration.

To demonstrate the efficacy of the batched kernels proposed in this work, Figures 5, 6 illustrate the capability of these kernels to efficiently exploit the resources of the device. These figures present the efficiency of the batched kernels in two regimes. The SM efficiency (Figure 5) illustrates the efficiency of the kernels at the SM level by calculating the percentage of time each SM has at least one active warp executing instructions. The warp execution efficiency (Figure 6) illustrates their efficiency at the warp level by calculating the percentage of active threads within each warp in the issuance of any particular instruction in the kernel execution. Deviations from 100% in the SM efficiency indicate that the SM is sitting idle due to some sort of contention, e.g. warp divergence, while deviations in the warp execution efficiency indicate that some warps have diverged such that the SM is only able to execute instructions to some subset of the threads within these diverged warps, reducing overall parallel efficiency. These performance measurements were obtained by the nvprof profiler metrics sm_efficiency and warp_execution_efficiency, respectively. As we can see, both the MAGMA provided batched BLAS and the hand optimized XC integration kernels developed for this work are able to achieve high SM efficiency, i.e. the SM is occupied and issuing instructions a high percentage of the time. With the exception of the SSF weights kernel, each of the batched kernels also exhibits an excellent warp execution efficiency (>90%), which means that there are not typically a large number of warp divergences in the execution of these kernels. The relatively low (60–70%) warp execution efficiency of the SSF kernels is due to the screening of weight partitions by the SSF scheme, i.e. adjacent quadrature points often follow different branch logic in the screening procedure. Note that the high SM and warp execution efficiencies for the kernel responsible for the batched evaluation of **Φ**^*j*^ by the simple method proposed in this work, combined with its relatively low cost percentage (>20%) for all problems considered, indicate that further optimization of this kernel by more advanced techniques would likely not yield a large impact on overall wall time.

### 3.2. Strong Scaling

**Figure 7:**
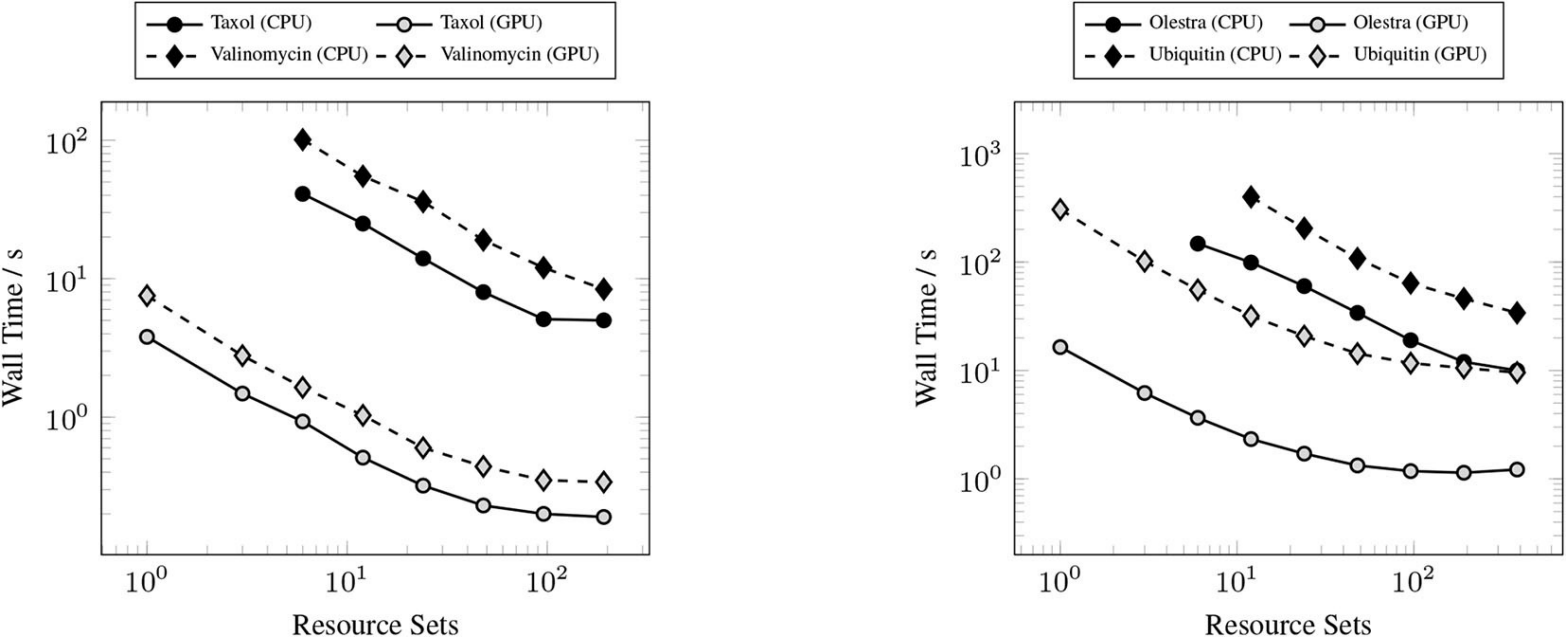
Strong scaling comparisons for the CPU (NWChem) and GPU (NWChemEx) implementations of the XC integration. Timings for both NWChem and NWChemEx include all steps in the XC integration (batch generation, weight scaling, local integration, and reduction).

**Figure 8:**
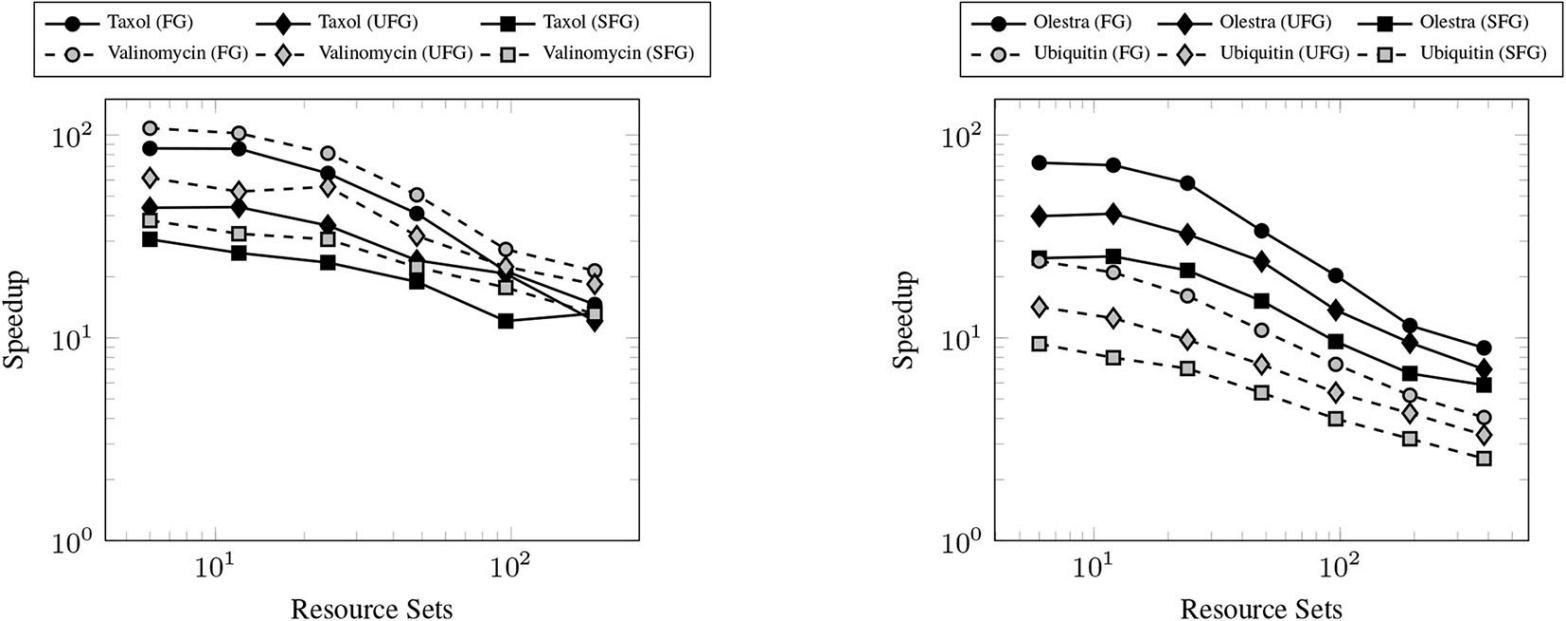
Achieved speedups of the GPU (NWChemEx) implementation over the CPU (NWChem) implementation of the XC integration for the 6-31G(d) basis set.

The primary goal of this work has been to provide a scalable implementation of the XC integration. As such, we examine the strong scaling the proposed method in comparison with the CPU implementation in NWChem. Strong scaling results for the CPU and GPU XC integrations using the 6-31G(d) basis and UFG quadrature are given in Figure 7. The wall times presented in Figure 7 only include those operations that are required to perform the XC integration; wall times for the allocation of device memory in the NWChemEx results have been removed. For the smallest problems (Taxol and Valinomycin), both NWChem and NWChemEx exhibit near linear strong scaling out to 4 Summit nodes (168 MPI ranks in the case of NWChem, and 24 GPUs in the case of NWChemEx). For largest problems (Olestra and Ubiquitin), linear strong scaling is exhibited up to 8 Summit notes (48 GPUs) in the case of NWChemEx and 16 nodes (336 MPI ranks) in the case of NWChem. The relative speedups of NWChemEx over NWChem for the considered systems in the 6-31G(d) basis set are given in Figure 8. For all but the largest problem (Ubiquitin), speedups over 10x are observed over the CPU implementation at all resource set counts. For the smallest problems with the smallest grid size (FG), speedups of ~100x are observed when run on a small number of resource sets. The degradation in speedup as a function in quadrature size is due to the aforementioned differences in weight and density screening techniques between NWChem and NWChemEx. The magnitude of these speedups decrease as the amount of resources increase. This is especially the case for ubiquitin, where a speedup of ~10x is observed at a single Summit node, but this speed up falls to nearly 2.5x in the strong scaling limit. To better understand the stagnation of strong scaling in this case, it is necessary to examine the scaling of the individual components of the XC integration.

**Figure 9:**
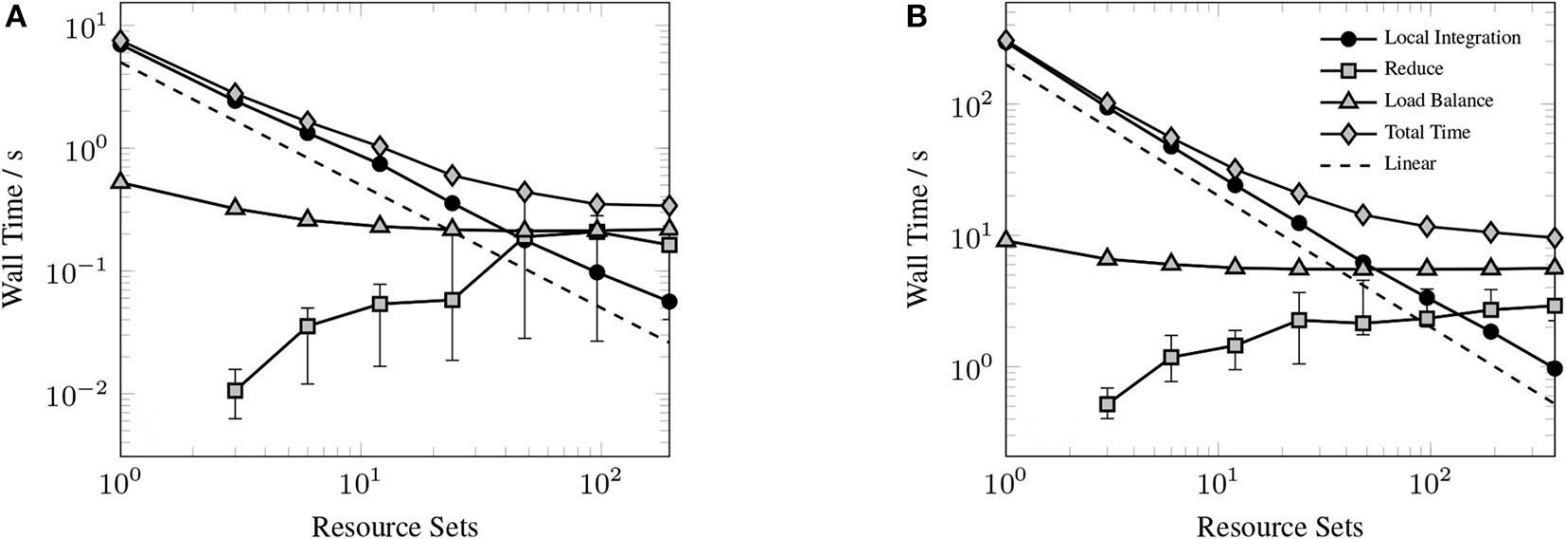
Strong scaling of individual components of the XC integration for valinomycin **(A)** and ubiquitin **(B)** in comparison to total execution time. Error bars represent min/max times and solid markers represent average wall time over all resource sets.

Figure 9 shows the timings for various components of the GPU XC integration for considered systems. Rather than examine the scaling for each of the considered systems, we choose to profile the largest of the small sized problems (Valinomycin), and the largest problem (Ubiquitin) as representative test cases. As can be seen in Figure 9, the local integration scales linearly for all processor counts considered. As the local integration scales linearly, stagnation is not due to a lack of sufficient work to occupy the GPU, but rather due to the increasing cost of the MPI reduction and the constant cost of replicating Algorithm 3 on all resource sets. This scaling behavior could be further improved by porting Algorithm 3 to the GPU, however, in the case of large processor counts, the reduction becomes competitive with Algorithm 3, thus it would be unlikely to demonstrate any qualitatively different scaling behavior in this regime.

## 4. Conclusion

In this work, we have proposed and implemented a three-level, GPU-based parallelism scheme for the distributed numerical integration of the XC potential and energy required for the evaluation of the Fock matrix in the Gaussian basis discretization of KS-DFT. In addition to the development of a simple load balancing scheme, the method proposed in this work for the evaluation of local integration quantities emphasizes the use of batched kernel invocations to achieve high throughput in the evaluation of localized quadrature batches on the GPU. This approach was motivated by the recent advent of GPU-accelerated batched BLAS kernels, which have seen wide adoption in many GPU applications. We have demonstrated that the proposed load balancing scheme produces linear strong scaling in the local integration of XC quantities for the problems considered. Further we have validated the efficacy of the use of batched kernels, including the use of batched GEMM and SYR2K, by demonstrating the ability of these kernels to achieve excellent efficiency on the NVIDIA Tesla V100 for a wide range of molecular systems, basis sets, and quadrature sizes.

The largest deficiency in the current work is the restricted implementation of the GPU-related techniques to NVIDIA GPUs and the CUDA SDK. As of this work, emerging architectures are increasingly relying upon other GPU vendors (AMD, Intel, etc.), which would render direct application of the current implementation impossible. However, the principles of batched kernel evaluation may be extended to many if not all GPU devices. Thus, as has been explored in the context of related implementations of seminumerical exchange calculations (Laqua et al., 2020), future work will focus on the *portable* implementation of the scalable GPU method presented in this work.

We have implemented the proposed method in the open-source NWChemEx software package and have demonstrated speedups between 10x and 100x over the analogous CPU implementation in NWChem. However, in the strong scaling limit, the proposed replicated load balance scheme and distributed reduction of XC integrands become computationally dominant, which causes early stagnation relative to the linearly scaling local integration on the GPU. As has been demonstrated in related work (Manathunga et al., 2020), porting the batch generation and screening procedure to the GPU would help mitigate the strong scaling stagnation, though the asymptotic bottleneck of the distributed reduction would still remain. With the one-to-one CPU-to-GPU affinity discussed in this work, the computational cost of the MPI reduction could be reduced through the use of remote memory access (RMA) to exploit shared memory spaces and void explicit data communication. As the local integration scales linearly out to very large processor and GPU counts, further improvements in these non-GPU aspects of the XC integration would drastically improve the strong scaling of the proposed methods. Such improvements will be explored in future work.

## Data Availability Statement

The original contributions presented in the study are included in the article/Supplementary Material, further inquiries can be directed to the corresponding author/s.

## Author Contributions

The software implementation of the purposed XC integration method is due to DW-Y. The algorithmic development of the load balance and distributed parallelism schemes is due to DW-Y, WJ, and CY. The development of software for the evaluation of the XC functional on the device as well as selection of the molecular test systems is due to DW-Y and HD. All authors contributed to the article and approved the submitted version.

## Conflict of Interest

The authors declare that the research was conducted in the absence of any commercial or financial relationships that could be construed as a potential conflict of interest.
